# Environmental footprints of food consumption and dietary patterns among Lebanese adults: a cross-sectional study

**DOI:** 10.1186/s12937-018-0393-3

**Published:** 2018-09-12

**Authors:** Farah Naja, Lamis Jomaa, Leila Itani, Jeremy Zidek, Sibelle El Labban, Abla Mehio Sibai, Nahla Hwalla

**Affiliations:** 1 Nutrition and Food Sciences Department, Faculty of Agriculture and Food Sciences, American University of Beirut, PO Box 11-0236, Riad El Solh, Beirut, 11072020 Lebanon; 2 Department of Nutrition & Dietetics, Faculty of Health Sciences, Beirut Arab University, PO Box 11-5020, Riad El Solh, Beirut, 11072809 Lebanon; 3 Department of Epidemiology and Population Health, Faculty of Health Sciences, American University of Beirut, PO Box 11-0236, Riad El Solh, Beirut, 11072020 Lebanon; 4 Futura Food LLC, 113 Creekside Drive, State College, PA 16801 USA

**Keywords:** Dietary patterns, Environmental footprint, Mediterranean, Sustainability, Lebanon

## Abstract

**Background:**

Following the release of the Sustainable Development Goals, dietary patterns and guidelines are being revised for their effect on the environment in addition to their health implications. The objective of this study was to evaluate and compare the Environmental Footprints (EFPs) of food consumption patterns among Lebanese adults.

**Methods:**

For this study, data for adults aged > 18 years (*n* = 337) were drawn from a previous national survey conducted in Lebanon (2008–2009), where dietary intake was assessed using a 61-item Food Frequency Questionnaire. Dietary patterns previously derived in the study sample included: Western, Lebanese-Mediterranean and High-Protein. In this study, food consumption and dietary patterns were examined for their EFPs including water use, energy use, and greenhouse gas (GHG) emissions, using review of life cycle analyses.

**Results:**

In the study population, the EFPs of food consumption were: water use: 2571.62 ± 1259.45 L/day; energy use: 37.34 ± 19.98 MJ/day and GHGs: 4.06 ± 1.93 kg CO2 eq / day. Among the three dietary patterns prevalent in the study population, the Lebanese-Mediterranean diet had the lowest water use and GHG per 1000 Kcal (Water (L/Kg): 443.61 ± 197.15, 243.35 ± 112.0, 264.72 ± 161.67; GHG (KG CO2 eq/day) 0.58 ± 0.32, 0.38 ± 0.24, 0.57 ± 0.37, for the Western, Lebanese-Mediterranean and High- Protein, respectively). The scores of the High-Protein dietary pattern were associated with higher odds of the three EFPs, whereas the Lebanese-Mediterranean dietary pattern was associated with lower odds of energy use. Furthermore, scores of the Western pattern were associated with higher water use.

**Conclusions:**

The findings of this study showed that, among Lebanese adults, the Western and High-Protein dietary patterns had high EFPs, whereas the Lebanese-Mediterranean dietary pattern had lower water use and GHG emissions. Coupled to our earlier findings of the Lebanese-Mediterranean pattern’s beneficial effects on health, the findings of this study lend evidence for the notion that what is healthy for people may also be healthy for ecosystems and highlight the need for nutrition recommendations to take into consideration the nexus of water, food, energy, in addition to health.

**Electronic supplementary material:**

The online version of this article (10.1186/s12937-018-0393-3) contains supplementary material, which is available to authorized users.

## Background

The Middle East and North Africa (MENA) region suffers from a high prevalence of non-communicable diseases (NCDs) constituting 47% of the region’s burden of diseases, and this rate is expected to rise up to 60% by 2020 [1]. Such a high burden of NCDs is possibly brought about by a shift in dietary habits and a remarkable nutrition transition [2]. This nutrition transition is characterized by a rapid shift in dietary intake from traditional, diverse and balanced diets towards more ‘westernized’ diets [1], specifically a high consumption of ‘harmful’ foods and low consumption of ‘protective’ foods [3]. This situation has triggered national and regional efforts to develop dietary guidelines and recommendations that promote the consumption of balanced diets known for their protective effects against NCDs [4, 5]. However, these efforts did not consider the environmental impact of such guidelines in a region that suffers of depleted resources, in terms of water scarcity, land degradation and high energy use [2]. These issues have been raised by the released Sustainable Development Goals (SDGs) which called for sustainable consumption and production and brought “sustainable diets” to the forefront of the sustainability agenda [6, 7]. ‘Sustainable diets’, according to the Food and Agriculture Organization (FAO) (2012), are *those diets with low environmental impacts that contribute to food and nutrition security and to healthy life for present and future generations. Sustainable diets are protective and respectful of biodiversity and ecosystems, culturally acceptable, accessible, economically fair and affordable; nutritionally adequate, safe and healthy; while optimizing natural and human resources* [8]. Accordingly, United Nations Environment Program (UNEP) (2016), called to reexamine food-based dietary guidelines, not only for their health outcomes but also with respect to their environmental sustainability, given the considerable impact of dietary choices and food consumption on the environment [9]. Efforts to quantify environmental impacts of food consumption led to the development of environmental footprint indicators, spanning multiple dimensions and including greenhouse gas emissions (carbon footprint), nitrogen release (nitrogen footprint), water use (blue and green water footprint) and land use (land footprint) [10].

Accordingly, a growing body of research explored the previously recommended diets and dietary patterns in terms of their impact on environmental footprints and depletion of natural resources [11, 12]. Results from these studies suggested that plant-based dietary patterns, which are primarily based on fruits, vegetables, legumes, whole grains, nuts and seeds, have a more positive impact on health and a lower environmental impact than animal-based dietary patterns. Nevertheless, these findings need to be considered through a context-specific lens to account for regional and local variations in agricultural practices, environmental resources, local food systems and cultural preferences of diverse populations [13].

Lebanon is a middle income country in the MENA region, which, similar to other neighboring countries, is witnessing an increase in the prevalence of diet-related diseases, a rapid nutrition transition coupled with scarcity and poor management of natural resources [1, 14]. Previous research in the country has studied dietary intake and food consumption patterns in terms of their effect on health and wellbeing. The results of these investigations consistently identified two main dietary patterns: ‘Western’ and ‘Lebanese- Mediterranean’, where, the Western pattern has been associated with adverse health outcomes, including obesity, hypertension, and metabolic syndrome. On the other hand, the Lebanese-Mediterranean pattern showed protective effects against metabolic abnormalities and type 2 diabetes [14–18]. These findings provided evidence for recommendations to limit consumption of the Western dietary pattern and encourage adherence to the Lebanese-Mediterranean diet in order to combat the rising epidemic of NCDs in the country. However, such dietary recommendations and guidelines were not examined in terms of their environmental sustainability, as called upon by the international community and the SDGs, in a country and a region where environmental issues are paramount. The aim of the present study was to evaluate the environmental footprints (EFPs) of overall food consumption and to examine the association of these EFPs with dietary patterns previously identified in Lebanon. Contributions of foods and food groups to the EFPs of each pattern were also examined.

## Methods

### Study design, participants and data collection

Data for this study were drawn from the cross-sectional National Nutrition and Non-Communicable Disease Risk Factor Survey (2008–2009). Details describing the sampling frame and techniques are described elsewhere [19]. In brief, the households which were considered as primary sampling units were selected, using a stratified cluster random sampling frame. The numbers of households from each cluster were proportional to the cluster’s size. Within each household, one adult with no history of chronic diseases (> 18 years of age) was randomly chosen and was invited to participate in the survey. At the participants’ homes, data collection was conducted following the WHO STEPwise approach to Surveillance (STEPS) [20] and included socio-demographic and lifestyle questionnaires, anthropometric measurements, biochemical assessment, as well as a food frequency questionnaire (FFQ) for the evaluation of dietary intake (*n* = 337) [21]. The study protocol was reviewed and approved by the Institutional Review Board of the American University of Beirut, and informed consent was obtained from all participants in the study.

For the purpose of this study, survey data pertaining to the participants’ socio-demographic and lifestyle characteristics as well as dietary intake were used. Socio-demographic characteristics included age, sex, crowding index, marital status, and education level. Crowding index (CI), defined as the average number of people per room, excluding the kitchen and bathroom, was used as a proxy for socioeconomic status, whereby a lower CI was associated with a higher socio economic status [22]. Earlier investigations showed that CI was associated with adherence to certain dietary patterns in Lebanon [19, 23, 15].The lifestyle factors considered in this study were related to smoking and physical activity. Physical activity was assessed using the short Arabic version of the International Physical Activity Questionnaire [24]. Using metabolic equivalents-mins per week, three levels of physical activity were determined (low, moderate, high). Dietary intake of study participants was assessed using a 61-item FFQ. These foods are listed in Additional file 1. This FFQ measured dietary intake over the 1 year preceding the interview. For each food item listed in the FFQ, a standard portion size was specified and five frequency choices were given (never, daily, weekly, monthly or yearly). This FFQ was designed by a panel of nutritionists and included culture specific dishes and recipes. It was tested on a convenient sample of Lebanese adults to check for clarity and cultural sensitivity. Further details about this FFQ are found elsewhere [15].

### Dietary patterns in the study population

Previous work by our research group derived dietary patterns prevalent among the survey participants (*n* = 337) [19, 21], whereby the 61 food items listed in the FFQ were grouped into 25 food groups based on similarities in ingredients, nutrient profile, and/or culinary usage [25] and were entered in the factor analysis. Food items having a unique composition (e.g. eggs, olives, and mayonnaise) were classified individually. Furthermore, certain food items such as falafel sandwiches, though made of legumes, were entered separately from this food group given that falafel is a fried form of legumes and the sandwiches are usually eaten out of home, unlike other legume-based dishes that are mainly consumed in stew form and prepared at home. The total intake for each group (expressed as daily gram intake) was calcualted by summing the daily gram intake of each food within the group. For example, the daily gram intake from the food group ‘Dried fruits’ was calculated by adding the daily gram intake of dried raisins, dried apricots and dried prunes. Further details about the derivation of these patterns are presented elsewhere [21]. In brief, three dietary patterns were identified using factor analysis: Western, Lebanese-Mediterranean, and High-Protein. The Lebanese-Mediterranean deitary pattern was previously proposed as a variant of the Mediterranean diet [26]. The factor scores for each of the identified pattern were calculated by multiple regression approach whereby independent variables in the regression equation are the standardized observed values of the food items in the estimated patterns. These predictor variables are weighted by regression coefficients, which are obtained by multiplying the inverse of the observed variable correlation matrix by the matrix of factor loadings. The factor scores are the dependent variables in the regression equation [27]. Each individual received a factor score for each dietary pattern. The obtained scores were normally distributed with a mean of zero and standard deviation equal 1. These scores indicated the degree to which each participant’s diet corresponds to the identified pattern. In this study, these scores were used to examine the association of dietary patterns with the EFP. The foods/ food groups that consituted these patterns and their factor loadings are described in Table 1.

**Table 1:** Food items/groups constituting the dietary patterns prevalent in the study population^ab^

Western	Lebanese-Mediterranean	High-Protein
Pizza, pies and refined grains (0.63)	Fruits (0.64)	Poultry (0.69)
Fast food sandwiches(0.57)	Legumes (0.56)	Meat (0.63)
Sweets (0.53)	Whole dairy products (0.53)	Fish (0.59)
Regular soda (0.51)	Olives (0.47)	Low fat dairy products (0.55)
Mayonnaise (0.45)	Vegetables (0.45)	Hot drinks (0.35)
Nuts and Seeds (0.43)	Burghol (whole wheat parboiled and crushed) (0.34)	Breakfast cereals (0.22)
Eggs(0.4)	Dried fruits (0.29)	Light soda (0.22)
Fats and oils(0.37)	Traditional suits (0.25)	
Ice cream(0.31)		
Bottled fruit juices(0.23)		
Alcoholic beverages (0.21)		

^a^Factor loading of the various food groups/items are presented in ()

^b^The dietary patterns and the food items-and their factor loading- making up these patterns were taken from data of Matta et al. (2016) [21]

### Derivation of environmental foot prints (EFPs)

For the purpose of this study, metrics for three EFPs were calculated including water use, energy use and GHGs (Additional file 1). The mapping of dietary patterns to their associated EFPs required the estimation of metrics for the EFPs of each of the 61 items listed in the FFQ which made up the food groups of these patterns. These metrics were estimated based on a review of previously published Life Cycle Assessment (LCAs) analyses. LCA is an internationally recognized method employed to estimate the impact of resource use at all stages of a product’s existence from creation to end of life [25]. The LCAs reviewed in this study followed the ISO 14040 and 14,044 [28]. LCAs conducted in Mediterranean or neighboring countries with similar climates to Lebanon were prioritized over LCAs conducted in countries of other regions in the world. For each EFP metric, the origins of LCA data (local, regional or international) used in its estimation are described in Additional file 2. In this appendix, the references used in estimating the EFPs for each food item are also listed. For each food item, LCAs that covered processes from cradle to market (or distribution point) were selected. LCAs that included use phase impacts (such as cooking, heating or refrigeration) were not considered in this study. In this study, the functional unit considered was 1.0 kg of food consumed. The functional unit is defined as a representative, reference measure of the studied system to which all inputs and outputs can be related [29]. For wine/beer, some LCAs report results in liters instead of Kg. Results in these studies were recalculated to a per kg basis assuming a density of 1.0 kg/l. For milk and milk products, though most LCAs report their results in Kg of milk, the percentage fat and protein in the milk could vary. Therefore the outcomes of the LCAs used for milk were adjusted to reflect the standard fat and protein corrected milk values of 3.5% fat and 3.2% and was used as fat-protein corrected milk (FPCM), a common practice in dairy LCAs, using the following formula: FPCM(kg) = 0.149 * milk kg + 5.89 * fat kg. + 3.48 * protein kg [30].

#### Water use

The water use environmental metric consisted of the total water use in liters (blue and green water combined) per kilogram of food consumed. Two important elements were considered in the calculation of the water use metric:

1. Consideration of the domestically produced vs imported proportion of each foods: The Food and Agriculture Organization (FAO) data [31] and the United Nations Comtrade database [32] were used to identify foods that are produced locally versus those that are imported. In addition, for imported foods, we have considered the top two countries by amount from where a certain food is imported.
2. Use of a water stress-based impact assessment method: Following step (1), water use was adjusted for each country using the water stress index (WSI) developed by Pfister et al., 2009 [33].WSI is considered an impact assessment component that allows accounting for crop production in water stressed areas.

In light of these two aforementioned considerations, the water use metric estimation in this study was adjusted using the below formula:

$$ \mathrm{Water}\ \mathrm{use}\ \left(\mathrm{adjusted}\right)=\left(\mathrm{Water}\ {\mathrm{use}}^{\ast}\%{\mathrm{produced}}^{\ast }\ \mathrm{WSILebanon}\right)+\left(\mathrm{Water}\ {\mathrm{Use}}^{\ast}\%{\mathrm{importedTotal}}^{\ast}\%{\mathrm{importedCountry}1}^{\ast }\ \mathrm{WSICountry}1\right)+\left(\mathrm{Water}\ {\mathrm{Use}}^{\ast}\%{\mathrm{importedTotal}}^{\ast}\%{\mathrm{importedCountry}2}^{\ast }\ \mathrm{WSICountry}2\right) $$

#### GHGs

The GHG metric was calculated in kg CO2 eq/kg food consumed. Most LCAs used in this study reported GHG emissions in terms of CO2 eq. However, a few LCAs reported CH_4_ and N_2_O separately, in addition to CO_2_. For these LCAs, emissions from N_2_O and CH_4_ were converted to kg CO2 eq using the following two equations [34]:

$$ {\mathrm{CO}}_{2\mathrm{eqN}2\mathrm{O}}={\mathrm{X}}_{\mathrm{N}2\mathrm{O}}\ast {\mathrm{GWP}}_{\mathrm{N}2\mathrm{O}} $$

$$ {\mathrm{CO}}_{2\mathrm{eq}\ \mathrm{CH}4}={\mathrm{X}}_{\mathrm{CH}4}\ast {\mathrm{GWP}}_{\mathrm{CH}4} $$

where X _N2O_ is the amount of N_2_O released in kg, X_CH4_ is the amount of CH_4_ released in kg, GWP _N2O_ is the 100-year global warming potential of N_2_O, and GWP_CH4_ is the 100-year global warming potential of CH_4_, GWP _N2O_ = 265, and GWP_CH4_ = 28 [34].

The total CO2 eq was calculated by adding CO_2eq N2O_ and CO_2eq CH4_ to the CO_2_ emissions. It is important to note that CH4 emissions from decomposing organic waste in landfills was not directly considered in this analysis due to a lack of specific data for each food item. Fluorinated gases are also not considered as their contributions to the accumulated GHGs of food products may be considered negligible [35].

#### Energy use

In this paper, ‘energy use’ referred to industrial energy consumption while ‘energy intake’ referred to human energy consumption. Energy use was estimated in MJ/kg food consumed. For all foods considered in this study, energy values and GHG emissions were sourced separately.

### Statistical analysis

Socio-demographic characteristics of the study population were described using proportions. For each food item listed in the FFQ, the EFPs metrics corresponding to each participant’s dietary intake was estimated using the below formula:

$$ \sum \limits_{i=1}^n consume{d}_i\ast impac{t}_i $$

where *n* is the food item number, *consumed*_*i*_ is the amount of food consumed in kg- as obtained from the FFQ, and *impact*_*i*_ is the EFPs per unit kg consumed for each food. The EFPs per unit Kg of food item are presented in Additional file 1. For example, the ‘rice and rice products’ food item is estimated to have a GHG impact of 2.05 kg CO_2eq_ / kg_consumed_. Therefore, if a survey participant consumed an average of one serving of rice (75 g) per day, then rice would contribute 0.15 kg CO_2eq_ to the overall GHG footprint of the diet of this participant. Accordingly, for each of the 61 food items, study participants had estimated metrics for the 3 EFPs that corresponded to their dietary intake (Additional file 1). The Means ± SD of EFP metrics for water use, energy use and GHGS were calculated for the overall dietary intake in the study population. In addition the EFPs of each of the three patterns were calculated by adding the EFPs of the food groups making up these patterns. To adjust for dietary energy intake, the EFPs for the various dietary patterns were also reported per 1000 Kcal and were compared using repeated measure ANOVA. The effects of adherence to any of the dietary patterns on the odds of having a high EFPs were estimated using multiple logistic regression models. For each dietary pattern, three separate regression models were built, each model corresponding to one of the three EFP metrics considered in this study. In each of these models, the independent variable was the score of one of the dietary patterns and the dependent variable was high EFPs. The latter was defined as belonging to the top 20% of the respective EFPs. These models were adjusted for age, sex and energy intake.

## Results

Table 2 describes the characterstics of the study popualtion, in terms of sociodemographic, lifestyle and dietary intake. Over 60% of the study population had a crowding index greater than 1 and were married. As for education, 34% of the study sample had a university level. Mean dietary energy intake was 2638.87 ± 1231.08 Kcal, with carbohydrates, proteins and fats contributing 49.06%, 15.71% and 36.48% respectively. (Table 2).

**Table 2:** Characteristics of the study population (*n* = 337)^a^

	Study population (*n* = 337)
Demographic characteristics	
Age (years)	
18–30	111(32.9)
31–40	103(30.6)
≥ 41	123(36.5)
Sex	
Males	168 (49.8)
Females	169 (50.2)
Social and lifestyle characteristics	
Crowding index	
< 1	122(36.4)
≥ 1	213(63.6)
*p*-value	
Marital status	
Single	129(38.3)
Married	208(61.7)
Education	
Up to High school	231(68.5)
University level	106(31.5)
Smoking	
No	231(68.5)
Yes	106(31.5)
Physical activity level	
Low	122(36.2)
moderate	68(20.2)
High	147(43.6)
Dietary intake characteristics	
Energy intake (Kcal)	2638.87 ± 1231.08
Carbohydrates (% of total energy)	49.06 ± 7.03
Proteins (% of total energy)	15.71 ± 2.90
Fats (% of total energy)	36.48 ± 6.90

^a^Values in this table represent n(%) and mean ± SD for categorical and continuous variables respectively

Table 3 presents the EFP of dietary intake in the study sample, as a total and by dietary pattern. Overall dietary intake of survey participants has the following EFPs (mean ± SD): Water use: 2571.62 ± 1259.45 L/day; Energy use: 37.34 ± 19.98 MJ/day GHG: 4.06 ± 1.93 kg CO_2eq_/day.

**Table 3:** Environmental Footprints of dietary intake in the study sample: Total and by dietary pattern ^§^

	Total	Western	Lebanese-Mediterranean	High-Protein
Water use (L / day)	2571.62 ± 1259.45	1231.02 ± 937.23^a^	602.06 ± 330.70^b^	653.87 ± 452.92^b^
Water use (L/day) per 1000 Kcal	951.68 ± 216.26	443.61 ± 197.15^a^	243.35 ± 112.0^b^	264.72 ± 161.67^a^
Energy use (MJ / day)	37.34 ± 19.98	20.53 ± 17.50^a^	10.82 ± 6.27^b^	5.00 ± 4.41^c^
Energy use (MJ / day)/1000 Kcal	14.25 ± 5.76	7.55 ± 4.85^a^	4.60 ± 2.87^b^	2.09 ± 1.84^c^
GHG (KG CO2 eq/day)	4.06 ± 1.93	1.58 ± 1.23^a^	0.90 ± 0.56^b^	1.40 ± 0.99^c^
GHG (KG CO2 eq/day)/1000 Kcal	1.53 ± 0.51	0.58 ± 0.32^a^	0.38 ± 0.24^b^	0.57 ± 0.37^a^

^§^Values with different superscripts are significantly different at *p* < 0.05

Values with different superscripts (a, b) are significantly different

After adjustment for energy (/1000 Kcal), for both water use and GHGs, the Lebanese-Mediterranean pattern had the lowest EFP (*p <* 0.05), while no significant difference was noted between the Western and the High-Protein patterns. For energy use, the highest EFP was that of the Western diet, followed by the Lebanese-Mediterranean diet and the High-Protein, with significant differences between these patterns at *p* <0.05. (Table 3).

Table 4 shows the results of the multiple logistic regression of the effect of adherence to each of the dietary patterns on the odds of high EFPs, controlling for age, sex and dietary energy intake. For water use, adherence to both the Western pattern and High-Protein dietary patterns was associated with higher odds of having a high water use footprint, while no association was noted with the Lebanese-Mediterranean dietary pattern. The magnitude of the association between a high EFP of water use with the Western dietary pattern was higher than that with the High-Protein dietary pattern (OR: 2.56 and OR 1.93 respectively). As for energy use, while the High-Protein dietary pattern was associated with higher odds of having high EFP for this metric (OR: 1.90, 95% CI: 1.00–4.00), a Lebanese-Mediterranean dietary pattern was associated with significantly lower odds (OR: 0.95, 95%CI: 0.92–0.98). For GHG, the High-Protein dietary pattern was significantly associated with having a high EFP for this metric (OR: 3.22, 95%CI: 1.96–5.28). (Table 4).

**Table 4:** Multiple logistic regression for the association between dietary patterns and the odds of high EFPs scores in the study population (*n* = 377) ^ab^

Dietary patterns	Water use (L / day)	Energy use (MJ / day)	GHG (kg CO2 eq / day)
Western	2.56 (1.13–5.18)	0.96 (0.93–1.00)	1.32 (0.62–2.81)
Lebanese-Mediterranean	1.01 (0.97–1.04)	0.95 (0.92–0.98)	1.26 (0.86–1.85)
High-Protein	1.93 (1.26–2.96)	1.90 (1.00–4.00)	3.22 (1.96–5.28)

^a^High environmental foot print is defined as belonging to the top 20% of each footprint

^b^The logistic regression models were adjusted for age, sex, and energy intake

Table 5 presents the percent contributions of various food or food groups to the 3 EFPs of each of the dietary patterns. The total EFPs of each pattern was based on the sum of EFPs of food groups making up this pattern. Within the Western pattern, the highest contributor to all EFPs was ‘All grains’ (water use: 22.67%; energy use: 32.80% and GHGs: 29.22%). For the Lebanese-Mediterranean dietary pattern, compared to other food/food groups making up this pattern, whole dairy products had the highest percentage contribution to water use (43.01%). ‘Vegetables’ contributed most to energy use (60.12%) and GHG (50.75%) of this pattern. Within the High-Protein pattern, the highest contributor to all three EFPs was ‘Meat’ (water use: 69.30%, energy use: 50.87% and GHG: 73.08%). (Table 5).

**Table 5:** Percentage contribution (mean ± SD) of food/food groups to EFP metric of the dietary patterns

Dietary patterns	Water use (L / day)	Energy use (MJ / day)	GHG (kg CO2eq / day)
Western			
Pizza, pies and refined grains	25.88 ± 14.39	5.88 ± 5.39	4.11 ± 3.87
Fast food sandwiches	11.59 ± 10.98	5.06 ± 6.25	21.62 ± 18.02
Sweets	14.67 ± 14.64	6.89 ± 7.36	5.75 ± 5.97
Regular Soda	8.26 ± 11.26	10.68 ± 14.03	4.81 ± 7.24
Mayonnaise	0.39 ± 0.82	0.34 ± 0.75	0.37 ± 0.85
Nuts and seeds	10.83 ± 13.36	0.93 ± 2.18	0.96 ± 2.02
Eggs	4.84 ± 7.66	1.47 ± 4.71	5.42 ± 7.91
Fats and oils	9.62 ± 8.01	12.24 ± 9.18	6.97 ± 6.35
Ice cream	0.32 ± 0.44	1.32 ± 1.79	1.98 ± 2.67
Bottled fruit juices	13.18 ± 17.30	21.31 ± 24.43	16.37 ± 20.40
Alcohol beverages	0.43 ± 2.23	1.08 ± 3.58	2.43 ± 6.90
Lebanese-Mediterranean			
Fruits	20.69 ± 14.09	10.62 ± 9.19	10.23 ± 8.73
Legumes	13.11 ± 9.59	0.81 ± 0.93	6.90 ± 6.39
Whole dairy products	43.01 ± 19.05	21.04 ± 18.73	22.02 ± 20.32
Olives	8.39 ± 11.33	3.27 ± 5.79	6.55 ± 10.04
Vegetables	10.29 ± 7.68	60.12 ± 20.49	50.75 ± 21.08
Burghol (whole wheat parboiled and crushed)	2.13 ± 4.42	3.18 ± 5.01	2.16 ± 3.81
Dried fruits	0.29 ± 1.06	0.36 ± 1.27	0.33 ± 1.19
Traditional sweets	2.09 ± 2.89	0.60 ± 0.92	1.07 ± 1.55
High-Protein			
Poultry	19.21 ± 14.27	18.21 ± 12.48	14.98 ± 12.44
Meat	69.30 ± 21.29	50.87 ± 22.73	73.08 ± 20.79
Fish	3.22 ± 7.76	19.50 ± 17.89	6.86 ± 11.06
Low fat dairy products	4.88 ± 12.31	6.02 ± 16.59	3.56 ± 11.58
Hot drinks	0.55 ± 2.17	0.03 ± 0.10	0.01 ± 0.03
Breakfast cereals	0.38 ± 5.45	0.56 ± 5.52	0.35 ± 5.45
Light Soda	2.47 ± 8.86	4.80 ± 14.15	1.16 ± 6.23

## Discussion

The results revealed comparable levels of water use, energy use, and GHG emission for food consumption of Lebanese adults to estimates of other countries. Furthermore, comparing dietary patterns, the Lebanese- Mediterranean diet had the lowest EFPs for water use and GHGs. In terms of associations between adherence (higher scores) to various dietary patterns and having a high EFP (belonging to the top 20% of the population), the results of this study suggested that adherence to either the Western or the High-Protein led to an increase in the odds for water use, the High-Protein pattern was also associated with higher odds for energy use and GHGs, while the Lebanese-Mediterranean was associated with lower odds for energy use.

For comparison purposes of the EFP of the Lebanese food consumption to other countries, the EFPs were calculated based on an intake of 2500 kcal/person. For water use, in this study, the average per-person water use (2451 L/day) was slightly lower than the global average (2799 L/day [36] and similar to estimates obtained for Finland (2377 L/day) [37]. Water use of food consumption in the United States and Italy had higher estimates (3998 L/day and 3469 L/day, respectively) [37]. Though within range, the water use estimated for average food consumption in Lebanon ought to be taken into consideration especially in view of the scarcity of natural resources. Lebanon together with other countries of the MENA region are among the most water stressed areas of the world, whereby the water availability per person is more than six times below the global average (1383 m^3^ to 8462 m^3^) [38]. The within-range estimate for water use associated with food consumption in this study coupled with water scarcity in the country is alarming in view of the high water cost of agricultural production, especially that the forecasted climate change is expected to further reduce rainfall by 6–8%, snow cover by 40%, and prolong drought periods for every 1 °C of temperature rise [39]. Regarding energy, the estimate obtained in this study (35 MJ/day) is higher than that of the United States (28 MJ/day) [40], this finding is alarming especially that Lebanon relies almost solely on imported energy, whether in the form of gas or oil, while its average citizen consumes greater kWh as compared to global estimate (3563 kWh in Lebanon vs 3030 kWh per person globally) [41]. As for GHGs associated with food consumption in Lebanon, the results of the study revealed an estimate (3.9 Kg CO2 eq/day) that is similar to other Mediterranean countries such as Greece (3.6 Kg CO2 eq/day) [42] and to the United States (3.56 Kg CO2 eq/day) [43] and lower than estimates reported in France (4.8 Kg CO2 eq/day) [44]. Such differences could be explained by variations in processes of agricultural practices/food production [45, 46] or composition of food consumption. Regarding the latter, Mediterranean countries tend to consume lower meat and meat products (main contributors to GHGs) as compared to Europe and the Unites States [47]. Despite the lower GHGs associated with food consumption in some Mediterranean countries (including Lebanon) as compared to Western countries, the recent review of the link between global diets, environmental sustainability and health suggested that adherence to a typical Mediterranean diet results in even lower estimate for GHGs (2.6 Kg CO2 eq/day), lending evidence for future research for dietary intake recommendations to lower the GHG associated in Mediterranean countries. Such research ought to consider the evaluation and implementation of dietary recommendation within the tightly linked diet–environment–health trilemma [48].

In this study, the results indicated that the Lebanese-Mediterranean dietary pattern had the lowest levels of water use and GHG emissions, as compared to the Western and the High-Protein patterns. The lower EFPs of the Lebanese-Mediterranean diet were also observed for other traditional diets in Japan [49], South Asia [50] and among indigenous people in Italy [51]. This finding is also in line with those of a previous investigation of the EFPs of dietary patterns in Spain which showed that adherence to a Western dietary pattern led to a significant increase in GHG, agricultural land use, energy as well as water consumption, while adherence to the Mediterranean diet reduced the impact on all EFPs metrics considered in that study [52]. Furthermore, a recent systematic review of studies examining dietary patterns and their environmental sustainability concluded that adherence to Mediterranean style diets has a less negative impact on the environment than current average dietary intakes in the United States [12]. In fact, within the definition of sustainable diets, the FAO and *Biodiversity International* in collaboration with other organizations acknowledged the Mediterranean diet as an example of sustainable diets with four main themes which related not only to health and wellbeing, but also to environment and sustainability, economy as well as well as culture [53, 54]. It is important to note that the Lebanese-Mediterranean shares many characteristics of the Mediterranean diet including high consumption of fruits, vegetables, and olive oil and olives. Hence the Lebanese- Mediterranean was previously proposed as a variant of the Mediterranean diet from the eastern side of the Mediterranean basin [26]. Similar to the other Mediterranean and traditional diets, the lower EFPs of the Lebanese-Mediterranean diet observed in this study could be due to the fact that limited intake of animal food and higher consumption of plant foods (vegetables, cereals and legumes), which are characteristics of this diet, have lower GHG emissions [55, 56]. The present study’s findings that the Lebanese-Mediterranean diet had lowest water and GHGs footprints, while the Western dietary pattern had high EFPs are of particular concern as the country is undergoing nutrition transition towards a more westernized dietary consumption. The ongoing nutrition transition would lead to intensification in EFPs and potentially impacting the available natural resources in a region facing significant water scarcity, climate, and landscape and ecosystems threats [53].

When the associations between the various patterns and EFPs were investigated, the results showed a direct association between a higher adherence with the High-Protein pattern and all three EFPs considered in this study. This finding echoes previous reports documenting that the consumption of animal-based food groups has the highest impact on the environment, including water use, energy use and GHGs [48].

In this study, the EFPs of dietary patterns were further characterized whereby the main contributors, in terms of foods and food groups, to the EFPs within these patterns were identified. Within the Western pattern, grains, fast food sandwiches and bottled juices contributed most to EFPs. The Fast food sandwiches of the Western pattern were composed of processed meat-based fillings, such as ‘Chawarma’ and hamburger. In many countries, red meat was identified as the greatest contributor to diet-related as well as overall agricultural GHG emissions [57–62]. In accordance with the results of this study, Hendrie et al. (2014) showed that red meat and non-core foods (which included processed meats, hot drinks and other energy-dense food items) in the Average Australian Diet accounted for the greatest contribution to GHG emissions [63]. The high contribution of bottled fruit juices to water use in the Western pattern, is in agreement with the reportedly high water footprint of apple and orange juice (200–230 L of water/200 ml glass or 1020–1140 L of water/L of juice) [64]. Interestingly in the Western pattern grains contributed most to the EFPs of this pattern. Although in general, grains are reported to be low on environmental impact [65], however, their high consumption by Lebanese adults led to their large contribution to the EFPs of this pattern. Within the Lebanese-Mediterranean pattern, dairy products alone contributed over 40% of the total water use of this pattern, 21% of energy use and 22% of its GHGs. Though not a common element of the Mediterranean diets, dairy products were part of the Lebanese-Mediterranean pattern. Lebanon, as well as other countries of the Levant including Syria and Jordan, traditionally consumes large quantities of dairy products, which are typically consumed as fermented milk products such as yogurt, strained yogurt (labneh) and white cheese in brine [66]. This finding is in line with other studies which showed that dairy products, principal sources of animal protein in some Mediterranean diets, presented high environmental footprints possibly due to the low consumption of meat and meat products in general in these diets [52, 67]. It is noteworthy that the vegetables group contributed to 60% of energy use and 51% of GHG in this pattern. These results could be explained by 1) the relatively high consumption of vegetables within the Lebanese diet and 2) the fact that production of vegetables requires more energy and GHGs than grains and fruits [48, 68]. In light of these findings, it appears sensible to recommend limiting dairy and increasing fruit consumption within the Lebanese-Mediterranean pattern. However future studies are needed to examine such recommendations taking into consideration the nexus of water, food, energy, as well as health. Policies focusing on one dimension of this nexus ignore the potential tradeoffs among the different impacts and may inadvertently strain resources [10]. For the High-Protein pattern, meat and poultry has the highest contribution to the EFPs of this pattern. As indicated earlier in this section, most research investigating the EFP of food consumption concluded that animal-based food groups, including meat and poultry pose the highest burden on the environment, including high water use, energy use and GHGs [48].

The strength of this study lies in it being the first in the MENA to examine EFPs of food consumption and dietary patterns and as such it could constitute a model for other countries in the region to emulate. The findings of this study ought to be considered in light of a few limitations. First, in the context of this study, metrics derived through the use of LCAs are to be interpreted with caution. LCAs allow for measuring the ecological footprint of each food or food category throughout its life cycle, which typically includes agricultural production and processing [12]. However most of the LCAs are conducted in high income countries with very few based in middle or low income countries. In this study, effort was exerted to use local and regional LCAs. If not available, LCAs from high income countries with similar climate and environmental conditions were used. Second, although this study addressed three EFPs, other elements of environmental sustainability such as soil erosion, biodiversity, pollution, farm management and ecosystem services were not considered in this study. It is important to note that the lack of a detailed inventory of agricultural and production practices in Lebanon and other countries of the region limit the feasibility of a comprehensive assessment of environmental footprints of food consumption.

## Conclusion

The findings of this study showed that, among Lebanese adults, food consumption in general and the Lebanese-Mediterranean dietary pattern had the lowest estimates for water and GHGs footprints, while the Western and High- Protein patterns had higher EFPs. Coupled to our earlier findings of the Lebanese-Mediterranean pattern’s beneficial effects on health and the Western pattern’s deleterious effects on health, the findings of this study lend evidence for the notion that what is healthy for people may also be healthy for ecosystems. These EFPs estimates associated with food consumption patterns in the country could be used to inform policies vis-a-vis agricultural production, type of production, food imports, subsidies and recommendations for sustainable food consumption. This study is a first step towards the formulation of sustainable diets for the Lebanese population. Further studies are needed to examine the nutrition value, quantity and quality of the food items comprising the identified patterns in order to achieve this goal and help countries address the SDGs.

## Additional files

Additional file 1: Environmental footprints of each of the 61 food items listed in the FFQ. (DOCX 22 kb)

Additional file 2: References used for the derivation of the EFPs by food item. (DOCX 31 kb)

## Abbreviations

CH4: Methane
CI: Crowding index
CO2eq: Carbon dioxide equivalent
EFP: Environmental Footprints
FFQ: Food Frequency Questionnaire
GHG: Greenhouse Gas
GWPCH4: Global warming potential of methane
GWPN2O: Global warming potential of nitrous oxide
ha: Hectar
LCA: Life cycle analysis
MENA: Middle East and North Africa Region
MJ: Mega Joules
N2O: Nitrous oxide
NCD: Non-Communicable Diseases
NRI: Natural Resources Inventory
SDG: Sustainable Development Goals
t: Metric Ton
UNEP: United Nations Environment Program
WHO: World Health Organization
