# Supplementary material for: Environmental footprints of food consumption and dietary patterns among Lebanese adults: a cross-sectional study
Source: Nutr J. 2018 Sep 12;17:85. doi: 10.1186/s12937-018-0393-3 (PMC6136176; doi:10.1186/s12937-018-0393-3)
Supplement: Supplementary file 1 — Environmental footprints of each of the 61 food items listed in the FFQ. (DOCX 22 kb) [file 12937_2018_393_MOESM1_ESM.docx]

Additional file 1 Environmental footprints of each of the 61 food items listed in the FFQ^*^

|  | Water Use  (L / kg) | Total water use  (L) | GHG (kg CO2eq / kg) | Total GHG  (Kg CO2eq) | Energy Use  (MJ / kg) | Energy use ( KJ) |
| --- | --- | --- | --- | --- | --- | --- |
| 1. Solid fat (butter, ghee) | 4915.25 | 32.45±54.49 | 6.04 | 0.04±0.07 | 90.00 | 0.59±1.00 |
| 2. Vegetable oil | 1971.66 | 54.10±31.95 | 1.56 | 0.04±0.03 | 44.00 | 1.21±0.71 |
| 3. Non wine alcoholic beverages | 1.29 | 0.00±0.02 | 0.92 | 0.00±0.02 | 3.52 | 0.01±0.06 |
| 4. Beer | 5.29 | 0.13±0.62 | 0.92 | 0.02±0.11 | 3.49 | 0.08±0.41 |
| 5. Wine | 866.96 | 4.10±17.42 | 2.89 | 0.01±0.06 | 19.00 | 0.09±0.38 |
| 6. Instant coffee | 117.47 | 20.11±24.20 | 0.35 | 0.06±0.07 | 1.00 | 0.17±0.21 |
| 7. Turkish coffee | 117.47 | 19.09±25.30 | 0.35 | 0.06±0.08 | 1.00 | 0.16±0.22 |
| 8. Cocoa (hot drink) | 8090.55 | 3.48±14.23 | 0.24 | 0.00±0.00 | 2.99 | 0.00±0.01 |
| 9. Bottled fruit juices | 2855.68 | 211.19±390.09 | 4.85 | 0.36±0.66 | 92.00 | 6.80±12.57 |
| 10. Fresh fruit juices | 572.89 | 30.69±44.58 | 0.75 | 0.04±0.06 | 11.90 | 0.64±0.93 |
| 11. Soft drinks | 626.99 | 131.22±386.71 | 0.43 | 0.09±0.27 | 13.71 | 2.87±8.46 |
| 12. Light soft drinks | 626.99 | 21.41±87.48 | 0.45 | 0.01±0.06 | 13.71 | 0.47±1.91 |
| 13.Olives | 4134.23 | 58.63±106.43 | 3.90 | 0.06±0.10 | 21.34 | 0.30±0.55 |
| 14. Mayonnaise | 2303.03 | 4.54±9.00 | 2.78 | 0.01±0.01 | 32.90 | 0.06±0.13 |
| 15. Low fat cheese | 4391.15 | 24.90±120.34 | 14.00 | 0.00±0.01 | 88.00 | 0.03±0.16 |
| 16. Cheese (full fat) | 4391.15 | 123.15±137.35 | 14.00 | 0.01±0.01 | 88.00 | 0.16±0.18 |
| 17. Milk (skimmed) | 547.74 | 9.58±36.21 | 1.50 | 0.06±0.24 | 31.73 | 0.56±2.10 |
| 18. Milk (full-fat) | 547.74 | 27.96±54.56 | 1.50 | 0.19±0.37 | 31.73 | 1.62±3.16 |
| 19. Labneh (strained yogurt) | 920.59 | 67.76±74.79 | 14.00 | 0.03±0.03 | 88.00 | 0.43±0.47 |
| 20. Yogurt (full fat) | 920.59 | 51.43±60.37 | 14.00 | 0.02±0.03 | 88.00 | 0.32±0.38 |
| 21. Yogurt (low fat) | 920.59 | 2.02±14.99 | 14.00 | 0.00±0.01 | 88.00 | 0.01±0.09 |
| 22. Nuts and seeds | 4942.69 | 149.11±213.14 | 0.42 | 0.01±0.02 | 5.00 | 0.15±0.22 |
| 23. Deep yellow orange fruits | 1119.16 | 26.91±37.76 | 0.42 | 0.01±0.01 | 5.00 | 0.12±0.17 |
| 24. Dried fruits | 3764.77 | 1.89±7.06 | 6.42 | 0.00±0.01 | 86.15 | 0.04±0.16 |
| 25. Bananas and apples | 684.72 | 58.57±66.01 | 0.42 | 0.04±0.04 | 5.50 | 0.47±0.53 |
| 26. Strawberry | 345.14 | 3.09±5.43 | 0.42 | 0.00±0.01 | 5.50 | 0.05±0.09 |
| 27. Citrus fruits | 511.12 | 26.06±31.95 | 0.42 | 0.02±0.03 | 5.00 | 0.25±0.31 |
| 28. Grapes | 504.14 | 8.67±10.54 | 0.42 | 0.01±0.01 | 5.50 | 0.09±0.11 |
| 29. Breakfast cereals | 484.32 | 0.36±1.30 | 0.86 | 0.00±0.00 | 19.5 | 0.01±0.04 |
| 30. Rice and rice products | 1332.84 | 91.51±77.28 | 2.05 | 0.14±0.12 | 19.09 | 1.31±1.11 |
| 31. Pasta | 521.34 | 11.11±19.34 | 1.33 | 0.03±0.05 | 17.77 | 0.38±0.66 |
| 32. Bulgur (Crushed wheat) | 584.10 | 10.72±16.41 | 0.86 | 0.02±0.02 | 19.50 | 0.30±0.45 |
| 33. Desserts^§^ | 484.32 | 4.13±7.14 | 1.02 | 0.01±0.02 | 13.75 | 0.10±0.18 |
| 34. Arabic sweets | 453.87 | 1.16±2.75 | 1.02 | 0.00±0.01 | 13.75 | 0.03±0.07 |
| 35. White bread | 507.97 | 97.85±87.36 | 0.86 | 0.17±0.15 | 19.50 | 3.10±2.77 |
| 36. Brown bread (whole wheat) | 507.97 | 10.36±23.59 | 0.86 | 0.02±0.04 | 19.50 | 0.33±0.75 |
| 37. Manaeesh^π^ | 507.97 | 30.12±40.75 | 0.86 | 0.05±0.07 | 19.50 | 0.95±1.29 |
| 38. Pizza | 507.97 | 6.24±14.42 | 5.35 | 0.01±0.02 | 11.60 | 0.14±0.33 |
| 39. Luncheon meat | 8938.71 | 50.45±112.40 | 69.00 | 0.12±0.26 | 43.00 | 0.24±0.54 |
| 40. Sausages | 8938.71 | 18.51±58.40 | 69.00 | 0.04±0.13 | 43.00 | 0.09±0.28 |
| 41. Eggs | 2709.95 | 50.30±121.58 | 4.00 | 0.07±0.18 | 11.00 | 0.20±0.49 |
| 42. Fish | 1246.00 | 14.77±21.43 | 6.47 | 0.08±0.11 | 79.83 | 0.95±1.37 |
| 43. Meat | 8938.71 | 399.24±311.28 | 69.00 | 0.91±0.71 | 43.00 | 1.92±1.50 |
| 44. Offals | 7341.41 | 15.06±58.12 | 37.15 | 0.03±0.10 | 32.50 | 0.07±0.26 |
| 45. Poultry | 3256.51 | 108.58±100.02 | 5.30 | 0.18±0.16 | 22.00 | 0.73±0.68 |
| 46. Chocolate | 8897.74 | 189.60±255.09 | 3.08 | 0.07±0.09 | 51.38 | 1.09±1.47 |
| 47. Ice cream | 458.02 | 3.15±3.74 | 14.00 | 0.03±0.03 | 31.73 | 0.22±0.26 |
| 48. Honey, jam and added sugar | 631.91 | 6.88±7.62 | 0.71 | 0.01±0.01 | 8.00 | 0.09±0.10 |
| 49. Dark green yellow vegetables | 334.82 | 6.16±12.87 | 1.60 | 0.03±0.06 | 26.90 | 0.49±1.03 |
| 50. Legumes | 2072.51 | 72.97±63.63 | 1.50 | 0.05±0.05 | 20.26 | 0.07±0.06 |
| 51. Potato | 248.70 | 5.10±6.97 | 0.12 | 0.00±0.00 | 0.50 | 0.01±0.01 |
| 52. Potato chips | 1244.37 | 0.21±0.65 | 0.54 | 0.00±0.00 | 6.00 | 0.00±0.00 |
| 53. Fried Potato | 1244.37 | 0.54±0.67 | 0.54 | 0.00±0.00 | 6.00 | 0.00±0.00 |
| 54. Tomato | 22.03 | 2.11±1.62 | 1.20 | 0.11±0.09 | 16.04 | 1.53±1.18 |
| 55. Season Salad | 228.26 | 39.40±34.18 | 1.40 | 0.24±0.21 | 21.47 | 3.71±3.22 |
| 56. Corn and peas | 557.03 | 10.56±16.23 | 0.42 | 0.01±0.01 | 2.70 | 0.05±0.08 |
| 57. Zucchini and eggplant | 134.30 | 2.66±5.28 | 1.60 | 0.03±0.06 | 26.90 | 0.53±1.06 |
| 58. Cauliflower | 242.51 | 3.23±4.76 | 1.40 | 0.02±0.03 | 21.47 | 0.29±0.42 |
| 59. Falafel sandwiches | 3092.67 | 28.57±66.61 | 0.53 | 0.00±0.01 | 11.10 | 0.09±0.20 |
| 60. Shawarma sandwiches | 3485.02 | 49.29±105.28 | 40.43 | 0.20±0.43 | 24.89 | 0.33±0.70 |
| 61. Hamburger | 3868.52 | 73.39±122.95 | 34.93 | 0.20±0.34 | 31.25 | 0.56±0.94 |

FFQ: Food Frequency Questionnaire; GHG: Green House Gas.

‘Water Use (L)’, ‘GHG (kg CO2eq)’ and ‘Energy Use (MJ)’ refer to the Total water use (L), Total GHG (Kg CO2eq) and Total Energy use ( KJ) by daily gram consumed.

^*^The EFP of each food item was calculated as EFP/kg multiplied by the amount consumed in the study population

^§^Desserts include cakes, cookies, doughnuts and muffins

^π^Manaeesh is the Lebanese version of the pizza dough
