# Supplementary material for: Environmental footprints of food consumption and dietary patterns among Lebanese adults: a cross-sectional study
Source: Nutr J. 2018 Sep 12;17:85. doi: 10.1186/s12937-018-0393-3 (PMC6136176; doi:10.1186/s12937-018-0393-3)
Supplement: Supplementary file 2 — References used for the derivation of the EFPs by food item. (DOCX 31 kb) [file 12937_2018_393_MOESM2_ESM.docx]

Additional file 2 References (in parenthesis) used for the derivation of the environmental footprints by food item separated into regionally (Mediterranean) or internationally sourced.

|  | Regional sources | International sources |
| --- | --- | --- |
| Water use | non-wine alcoholic beverages (2)^#^, beer (2)^#^, instant coffee (25)  Turkish coffee (25), bottled fruit juices (19), fresh fruit juices (19), olives (5), breakfast cereals (6), desserts ( cakes, cookies, donuts, muffins) (6), zucchini and eggplant (20) | Solid fat (butter ghee) (10), vegetable oil (6), wine (9), cocoa (hotdrink) (6), soft drinks (6,15), light soft drinks (6,15), mayonnaise (6,10), low fat cheese (10), cheese (full fat)(10), milk skimmed (10), milk (full fat) (10), lebneh (strained yogurt)(10), yogurt (full fat) (10), yogurt (low fat) (10), nuts and seeds (6), deep yellow orange fruits (6), dried fruits (6)  banana and apples (6), strawberry (6), citrus fruits(6), grapes (6), rice and rice products (6), pasta (6,13), bulgur (crushed wheat) (6)  Arabic sweets (6), white bread (6), brown bread (whole wheat)(6)  Manaeesh (the Lebanese version of pizza) (6), pizza (6), luncheon meat (10), sausages (10)  eggs (10), fish (16), meat (10), offals (10), poultry (10), chocolate (6), ice cream (10), honey, jam, and sugar (10), dark green yellow vegetables (6), legumes (6), potato (6), potato chips (6), fried potato (6), tomato (3), season salad (6), corn and peas (6), cauliflower (6), falafel sandwich (6), chawarma sandwich (6), hamburger(6). |
| GHGs | Solid fat (butter ghee) (14), vegetable oil (14), non-wine alcoholic beverages (14), beer (14), wine (1), instant coffee (25), Turkish coffee (25), bottled fruit juices (19), fruit juices fresh (19), mayonnaise (4,14), nuts and seeds (14), deep yellow orange fruits (14), dried fruits (14), banana and apples (14), strawberry (14), citrus fruits (14)  grapes (14), pasta (14), desserts(cake, cookies, donuts, muffins) (14), Arabic sweets (14), honey, jam, and sugar (14), dark green yellow vegetables (20), potato (14), potato chips (14), fried potato (14), season salad (14), zucchini and eggplant (20), cauliflower (14), milk skimmed (29), milk (full fat) (29), luncheon meat (29), meat (29), offals (7, 29), ice cream (29), chawarma sandwiches (29,21), hamburger (29,21) | cocoa (hotdrink) (17), soft drinks (26), light soft drinks (26), olives (5), low fat cheese (31), cheese (full fat) (31), lebneh (31), yogurt (full fat) (31), yogurt (low fat) (31), breakfast cereals (21), rice and rice products (21), bulgur (crushed wheat) (21), white bread (21), brown bread (whole wheat) (21), Manaeesh (the Lebanese version of pizza) (21), pizza (31,21), sausages (29), eggs (4), fish (11), poultry (7), chocolate (18), legumes (4), tomato (3), corn and peas (27), falafel sandwiches (4,27) |
| Energy use | Solid fat (butter ghee) (14), vegetable oil (14), non-wine alcoholic beverages (2)^#^, beer (2)^#^, wine (14)^@^, instant coffee (25), Turkish coffee (25), bottled fruit juices (19), fresh fruit juices (19), olives (5), mayonnaise (7.14), nuts and seeds (14), deep yellow orange fruits (14), dried fruits (14), banana andapples (14), strawberry (14), citrus (14), grapes (14), breakfast cereals (14), rice and rice products (21,22), pasta (3,14), bulgur (crushed wheat)(14), desserts(cakes, cookies, donuts, muffins) (14), Arabic sweets (14), white bread (14), brown bread (whole wheat) (14), manaeesh (the Lebanese version of pizza) (6), pizza (14), pizza (4,14), honey, jam, sugar (14), dark green yellow vegetables (20), potato (14), potato chips (14), fried potato (14), season salad (14), zucchini and eggplant (20), cauliflower (14) | cocoa (hot drinks)(17), soft drinks (26), liht soft drinks (26), milk (skimmed) (28), milk (full fat) (28), luncheon meat (7), sausages (7,14), eggs (7), fish (11), meat (7), offals (7), poultry (7), chocolate (18), ice cream (28), legumes (30), tomato (3), corn and peas (27), falafel sandwiches (27,14), chawarma sandwiches (8,14), hamburger (7,14), low fat cheese (31), cheese (full fat) (31), lebneh (31), yogurt (full fat) (31), yogurt( low fat )(31) |
| ^#^ Assumed similar to beer in bottle for non-wine alcoholic beverages and an equal mixture of beer produced for keg and bottle sales.  @ assumed coffee but without the heavy processing component that coffee uses. | | |

| Study | | References used for the derivation of the EFPs by food item |
| --- | --- | --- |
| 1 | Rugani, B., Vázquez-Rowe, I., Benedetto, G., & Benetto, E. (2013). A comprehensive review of carbon footprint analysis as an extended environmental indicator in the wine sector. Journal of cleaner production, 54, 61-77. | |
| 2 | Cordella, M., Tugnoli, A., Spadoni, G., Santarelli, F., & Zangrando, T. (2008). LCA of an Italian lager beer. *The International Journal of Life Cycle Assessment*, *5*(2), 133-139. | |
| 3 | Page, G., Ridoutt, B., & Bellotti, B. (2012). Carbon and water footprint tradeoffs in fresh tomato production. *Journal of Cleaner Production*, *32*, 219-226. | |
| 4 | Nijdam, D., Rood, T., & Westhoek, H. (2012). The price of protein: Review of land use and carbon footprints from life cycle assessments of animal food products and their substitutes. *Food Policy*, *20*(6), 760-770. | |
| 5 | Avraamides, M., & Fatta, D. (2008). Resource consumption and emissions from olive oil production: a life cycle inventory case study in Cyprus. *Journal of Cleaner Production*, *16*(7), 809-821. | |
| 6 | Mekonnen, M. M., & Hoekstra, A. Y. (2011). The green, blue and grey water footprint of crops and derived crop products. *Hydrology and Earth System Sciences*, *6*(5), 1577-1600. | |
| 7 | De Vries, M., & De Boer, I. J. M. (2010). Comparing environmental impacts for livestock products: A review of life cycle assessments. *Livestock science*,*128*(1), 1-11. | |
| 8 | Jones, A. K., Jones, D. L., & Cross, P. (2014). The carbon footprint of lamb: sources of variation and opportunities for mitigation. *Agricultural Systems*,*123*, 97-107. | |
| 9 | Herath, I., Green, S., Horne, D., Singh, R., McLaren, S., & Clothier, B. (2013). Water foot printing of agricultural products: evaluation of different protocols using a case study of New Zealand wine. *Journal of cleaner production*, *27*, 159-167. | |
| 10 | Mekonnen, M. M., & Hoekstra, A. Y. (2012). A global assessment of the water footprint of farm animal products. *Ecosystems*, *6*(3), 401-415. | |
| 11 | Samuel-Fitwi, B., Nagel, F., Meyer, S., Schroeder, J. P., & Schulz, C. (2013). Comparative life cycle assessment (LCA) of raising rainbow trout (Oncorhynchus mykiss) in different production systems. *Aquacultural engineering*, *54*, 85-92. | |
| 12 | Zonderland-Thomassen, M. A., & Ledgard, S. F. (2012). Water footprinting–A comparison of methods using New Zealand dairy farming as a case study. *Agricultural Systems*, *110*, 16-23. | |
| 13 | Ridoutt, B. G., & Pfister, S. (2010). A revised approach to water footprinting to make transparent the impacts of consumption and production on global freshwater scarcity. *Global Environmental Change*, *10*(1), 113-120. | |
| 14 | Monforti-Ferrario, F., & Pascua, I. P. (Eds.). (2015). *Energy use in the EU food sector: State of play and opportunities for improvement*. Publications Office. | |
| 15 | Pacific Institute. Bottled Water and Energy Factsheet. 2007. Accessed at: http://pacinst.org/publication/bottled-water-and-energy-a-fact-sheet/ | |
| 16 | Pahlow, M., Van Oel, P. R., Mekonnen, M. M., & Hoekstra, A. Y. (2015). Increasing pressure on freshwater resources due to terrestrial feed ingredients for aquaculture production. *Science of the Total Environment*,*536*, 847-857. | |
| 17 | Ntiamoah, A., & Afrane, G. (2008). Environmental impacts of cocoa production and processing in Ghana: life cycle assessment approach. *Journal of Cleaner Production*, *16*(16), 1735-1740. | |
| 18 | Büsser, S., & Jungbluth, N. (2009). LCA of Chocolate Packed in Aluminium Foil Based Packaging. *ESU-services Ltd., Uster, Switzerland. Available online: http://www. alufoil. org/tl_files/sustainability/ESU_-_Chocolate_2009_-_Exec_Sum. pdf (accessed on 25 May 2015)*. | |
| 19 | Beccali, M., Cellura, M., Iudicello, M., & Mistretta, M. (2010). Life cycle assessment of Italian citrus-based products. Sensitivity analysis and improvement scenarios. *Journal of environmental management*, *91*(7), 1415-1428. | |
| 20 | Cellura, M., Longo, S., & Mistretta, M. (2012). Life Cycle Assessment (LCA) of protected crops: an Italian case study. *Journal of cleaner production*, *28*, 56-62. | |
| 21 | Roy, P., Shimizu, N., Okadome, H., Shiina, T., & Kimura, T. (2007). Life cycle of rice: Challenges and choices for Bangladesh. *Journal of food engineering*,*79*(4), 1250-1255. | |
| 22 | Blengini, G. A., & Busto, M. (2009). The life cycle of rice: LCA of alternative agri-food chain management systems in Vercelli (Italy). *Journal of environmental management*, *90*(3), 1512-1522. | |
| 23 | Food and Agriculture Organization of the United Nations. FAO Statistics Division FAOSTAT. 2015. Accessed at: http://faostat3.fao.org/home/E | |
| 24 | Sagar, V. R., & Kumar, P. S. (2010). Recent advances in drying and dehydration of fruits and vegetables: a review. *Journal of food science and technology*, *30*(1), 6-26. | |
| 25 | Humbert, S., Loerincik, Y., Rossi, V., Margni, M., & Jolliet, O. (2009). Life cycle assessment of spray dried soluble coffee and comparison with alternatives (drip filter and capsule espresso). Journal of Cleaner Production, 7(6), 1351-1358. | |
| 26 | Amienyo, D., Gujba, H., Stichnothe, H., & Azapagic, A. (2013). Life cycle environmental impacts of carbonated soft drinks. The International Journal of Life Cycle Assessment, 8(1), 77-92. | |
| 27 | Kim, S., Dale, B. E., & Jenkins, R. (2009). Life cycle assessment of corn grain and corn stover in the United States. The International Journal of Life Cycle Assessment, 14(2), 160-174. | |
| 28 | Upton, J., Humphreys, J., Koerkamp, P. G., French, P., Dillon, P., & De Boer, I. J. M. (2013). Energy demand on dairy farms in Ireland. Journal of dairy science, 96(4), 6489-6498. | |
| 29 | Opio, C., Gerber, P., Mottet, A., Falcucci, A., Tempio, G., MacLeod, M., ... & Steinfeld, H. (2013). Greenhouse Gas Emissions from Ruminant Supply Chains—A Global Life Cycle Assessment (Food and Agriculture Organization of the United Nations, Rome). | |
| 30 | Kim, S., & Dale, B. E. (2003). Cumulative energy and global warming impact from the production of biomass for biobased products. *Journal of Industrial Ecology*, *7*(3‐4), 147-162. | |
| 31 | Kim, D., Thoma, G., Nutter, D., Milani, F., Ulrich, R., & Norris, G. (2013). Life cycle assessment of cheese and whey production in the USA. *The International Journal of Life Cycle Assessment*, *8*(5), 1019-1035. | |
